# Determinants and outcomes of health-promoting lifestyle among people with schizophrenia

**DOI:** 10.1186/s12888-024-05625-2

**Published:** 2024-03-04

**Authors:** Yu Fan, Liang Zhou, Xiyuan Chen, Jinghua Su, Shaoling Zhong

**Affiliations:** 1 The Affiliated Brain Hospital of Guangzhou Medical University, 36 Mingxin Road, Liwan District, 510370 Guangzhou, Guangdong China; 2 Key Laboratory of Neurogenetics and Channelopathies of Guangdong Province and the Ministry of Education of China, Guangzhou Medical University, Guangzhou, China

**Keywords:** Schizophrenia, Healthy lifestyle, Loneliness, Physical activity, Metabolic syndrome

## Abstract

**Background:**

Healthy lifestyle is an important protective factor of developing cardiovascular disease in people with schizophrenia. However, little is known about the determinants of lifestyle and its contribution to metabolic syndrome. This study aimed to explore the influencing factors of health-promoting lifestyle (HPL) and its association with metabolic syndrome among people with schizophrenia.

**Methods:**

A cross-sectional study was conducted in twenty-two primary health centers of Guangzhou, China between December 2022 and April 2023. A total of 538 patients with schizophrenia were recruited through convenience sampling. Self-administered scales, questionnaires, and clinical data were collected. Scales and questionnaires included social-demographic information, Health-Promoting Lifestyles Profile (HPLP-C), UCLA Loneliness Scale (ULS), and International Physical Activity Questionnaire-Short Form (IPAQ-SF). Cluster analyses were used to divide participants into two groups based on the distribution characteristics of HPLP-C scores. Logistic regression models were used to identify factors associated with HPL and the association between HPL and metabolic syndrome.

**Results:**

There were 271 participants in the high HPL group and 267 participants in the low HPL group. Logistic regression analysis revealed that loneliness posed a risk factor for high HPL, while high education and moderate-vigorous physical activity served as protective factors for high HPL. Low HPL was a risk factor for the prevalence of metabolic syndrome.

**Conclusions:**

Promotion of high education literacy and a physically active lifestyle should be priority targets in the health management of schizophrenia. Primary healthcare providers can play a pivotal role in assisting patients to mitigate metabolic syndrome by reinforcing healthy lifestyle strategies.

**Supplementary Information:**

The online version contains supplementary material available at 10.1186/s12888-024-05625-2.

## Introduction

Schizophrenia is a severe mental disorder that affects approximately 1% of the world’s population [1]. Persons with schizophrenia are much more likely to have a shorter life expectancy than the general population [2]. Previous studies have indicated a strong association between cardiovascular diseases (CVDs) and premature death of patients with schizophrenia [3]. Among these conditions, metabolic syndrome (MetS), characterized by central obesity, high blood pressure, hyperglycemia, and dyslipidemia, emerges as a worthy-considering physical illness concern for people with schizophrenia [4, 5]. Numerous studies have demonstrated a 2- to 3-fold higher prevalence of MetS in people with schizophrenia compared to the general people [6]. The elevated prevalence of MetS has been recognized as the main risk factor for CVDs [7].

Unhealthy lifestyles are the primary cause of MetS. When compared with general population, patients with schizophrenia tend to have poorer dietary, lower physical activity levels and higher usage of tobacco and alcohol [8]. Factors contributing to unhealthy lifestyles may be attributed to the nature of the disease itself, treatment modalities, and social environment. The side effects of antipsychotic drugs, coupled with symptoms like amotivation, apathy, and cognitive deficits, pose challenges for patients in adopting healthy lifestyles [9]. In addition, patients who have lower education levels and socioeconomic status are prone to have unhealthy lifestyles [10]. Notably, patients with schizophrenia often experience high level of stigma and discrimination, exacerbating their social isolation [11] and thereby impeding the development of healthy habits. Several studies have demonstrated that modifiable factors, including poor nutrition, low physical activity level, and high body mass index (BMI) [12, 13] are important determinants of CVDs, besides some static factors, such as genetic vulnerability [14]. A longitudinal study conducted in the United States population found participants who had lower-risk lifestyles, such as non-smoking, restriction of alcohol consumption, keeping a healthy weight, positive physical activity and a balanced diet, tended to live longer [15]. Adopting a healthy lifestyle may alleviate the elevated rates of morbidity and mortality in patients with schizophrenia.

A health-promoting lifestyle (HPL) is a positive way of life that contributes to the development of a high health status. Walker and his colleagues have provided a comprehensive perspective on HPL [16], describing it as a multidimensional model of perceptions and actions, including health responsibility, physical activity, nutrition, interpersonal relations, spiritual growth, and stress management. Researchers have evaluated the level of HPL and its influencing factors in many different populations, such as older adults [17], university students [18], and postmenopausal women [19]. Some research have been conducted in people with physical diseases, such as cardiovascular and cerebrovascular diseases [20]. Risk factors for HPL in physical diseases typically include poor economic income, poor family support, physical inactivity, low education level, poor marital status, and loneliness [20–22]. However, there have been limited studies evaluating the level of HPL in people with schizophrenia [23], and exploration of its influencing factors is yet to be undertaken.

Studies on the prevalence of MetS in patients with schizophrenia are replete, while there is a scarcity of studies on examining the risk factors associated with MetS, particularly focusing on HPL. Previous studies have commonly focused on how poor dietary and negative physical activity contribute to MetS. However, the broader scope of HPL may be overlooked, which extends beyond the two factors. One study showed that MetS was related to HPL scores among people with chronic schizophrenia in inpatients settings [23]. To date, there is a dearth of literature examining HPL and the relationship to MetS in primary health settings.

Given the heightened risk of MetS and the critical role of healthy lifestyle, we explored the influencing factors of HPL in people with schizophrenia living in communities using a cross-sectional study design. We aimed to identify the factors associated with HPL in patients with schizophrenia and to explore the relationship between HPL and MetS.

## Methods

### Study design and participants sampling

We adopted a cross-sectional study design and applied convenience sampling to recruit patients with schizophrenia from twenty-two primary health centers in three districts in Guangzhou, China between December 2022 and April 2023. In accordance with China’s national basic public health service in China, patients with severe mental illness can take annual free physical examination at primary health centers. In this study, primary healthcare professionals, responsible for the mental health of these patients contacted and encouraged them to undergo the physical examination. Participants were recruited based on predefined inclusion and exclusion criteria. Upon completing the survey, participants were rewarded with complementary gifts, such as towel, umbrella and toothbrush, valued at forty yuan.

Inclusion criteria encompassed individuals who: (1) met diagnostic criteria for schizophrenia based on the 10th edition of the International Classification of Diseases (ICD-10); (2) were aged 18 to 65 years; (3) had a course of illness lasting one year or longer and were currently in a chronic state; (4) underwent physical examinations in primary health centers; (5) lived in communities for at least 6 months; (6) comprehended the study contents and provided the written informed consent. Exclusion criteria involved individuals with: (1) hearing and visual disturbances; (2) serious physical disease, such as cardiovascular disease; (3) pregnancy or lactation.

The MetS prevalence, the primary outcome of this study, was used to calculate the sample size required for the study. Specifically, the sample size was calculated by the formula for cross-sectional study: n = *Z*_*1−α/2*_^2^*p(1-p) /d^*2*^. We set the *p* as 24.5% based on a previous meta-analysis [24], and estimated a sample size of 284 people would be needed, with *Z* = 1.96 at a confidence interval of 95% and allowable error *d* as 5%. Then we assumed a 60% participation rate in physical examination and a 10% data missing rate. Consequently, the sample size was expanded to 526. We also calculated a minimal sample size for the HPL, the other primary outcome of this study. We ensured a robust sample size of 526 participants, as recommended by Bujang et al. [25]. The guideline suggests a minimum sample size of 500 for observational studies employing logistic regression. Consequently, we assumed that about 526 people will be recruited.

Ethical approval was obtained from the ethics committee of the Affiliated Brain Hospital of Guangzhou Medical University, and all study participants provided written informed consent prior to the survey. A total of 594 patients were initially recruited, with 56 excluded due to missing data, resulting in a final sample of 538 included for statistical analysis.

### Data collection and measures

Patients who agreed to participant were invited to complete a battery of paper-based questionnaires. The questionnaires were coded and verified by the research group. Clinical data including blood pressure, glucose, and lipid metabolism indicators of all participants were tested in primary health centers by primary healthcare professionals. The information was stored in Guangzhou Mental Health System and one researcher exported these data for the study.

We used a self-administered questionnaire to obtain social-demographic data including age, sex, marriage status, employment status, education level, tobacco and alcohol consumption, illness duration, weight, height, waist circumference, and use of antipsychotics. Education level was categorized with primary education and below (i.e., primary school and below), secondary education (i.e., junior high school, senior middle school, and vocational school) and higher education (i.e., bachelor and above). Antipsychotics was categorized with first generation antipsychotics only (FGA), second generation antipsychotics only (SGA), both FGA and SGA, and non-antipsychotics treatment.

### The Chinese version of health-promoting lifestyles profile (HPLP-C)

The 42-item HPLP-C is a self-rating instrument that is used to identify participants’ health-promoting lifestyles and has been widely used in China [26, 27]. Items are measured using a four-point Likert scale (1 = never, 2 = sometimes, 3 = often and 4 = routinely). The total score ranges from 42 to 168, with higher scores indicating healthier lifestyle choices. In this research, the Cronbach’s alpha of the scale was 0.970, indicating good reliability and validity.

### UCLA loneliness scale (ULS)

The 20-item ULS is a self-rating scale, which is used to measure the level of loneliness [28]. Each item is measured using a four-point Likert scale ranging from 0 (never) to 4 (always). Nine items of this scale are negatively worded, and their scores are reverse coded. The total score ranges from 0 to 80. A higher ULS score indicates a higher level of loneliness. In this research, the Cronbach’s alpha of the scale was 0.783.

### International physical activity questionnaire-short form (IPAQ-SF)

The IPAQ-SF is a self-administered questionnaire, which is used to measure individuals’ physical activity (PA) during the last seven days [29]. This questionnaire includes low-intensity activities, moderate-intensity activities, and vigorous-intensity activities. Participants were required to report the frequency and duration that they engaged in each intensity activity. The total PA per week for each participant was calculated and then divided into low, moderate, and vigorous three levels following the IPAQ methodology as referenced in previous researches [30, 31].

### Diagnostic criteria for metabolic syndrome (MetS)

According to the diagnostic criteria set by the Chinese Diabetes Society [32], MetS is diagnosed when an individual meets at least three out of five of the following conditions: (a) abdominal obesity: waist circumference (WC) ≥ 90 cm in male and ≥ 80 cm in female; (b) fasting blood glucose (FBG) ≥ 5.6 mmol/L or a previous diagnosis and treatment for diabetes; (c) hypertension: systolic blood pressure (SBP) ≥ 130 mmHg or diastolic blood pressure (DBP) ≥ 85 mmHg, or the use of specific medicine for hypertension; (d) fasting triglyceride (TG) ≥ 1.70 mmol/L or the use of specific medicine for lipid abnormalities; and (e) fasting high-density lipoprotein cholesterol (HDL-C) < 1.03 mmol/L in males and < 1.29 mmol/L in females, or the use of specific medicine for lipid abnormalities.

### Statistical analysis

All statistical analyses were conducted using SPSS 25.0. Incomplete data were not included in the final data analysis. Mean and standard deviation (SD) were used to describe continuous variables. Numbers and proportions were used to describe categorical variables. A cluster analysis was conducted across the HPLP-C total score using the K-means algorithm. The number of clusters was defined as two and the cluster membership was saved as the grouping variable. The *t-*test and *χ*^*2*^ test were performed for group comparisons of continuous variables and categorical variables, respectively. We used logistic regression model to explore risk factors (i.e., illness duration, loneliness level, PA level, education level, occupation status, and the use of antipsychotics) associated with HPL. We defined these risk factors as independent variables, while the group of HPL was set as the dependent variable. We also used logistic regression model to explore the relationship between HPL and MetS. In this analysis, risk factors (i.e., HPL, Age, gender, occupation, BMI, loneliness level, PA level and the use of antipsychotics) were defined as independent variables, while MetS was set as the dependent variable. All variables with *p* < 0.05 in the univariate analyses were included in the logistic regression models. The results of logistic regression were presented as odds ratios (*OR*) and 95% confidence intervals (*95% CI*). A two-sided *p* < 0.05 was considered statistically significant.

## Results

### Social-demographic and clinical characteristics of participants

A total of 538 patients with schizophrenia (253 male and 285 female) were enrolled in this study. The average age of the participants was 44.70 (SD = 11.45), with a mean illness duration of 16.91 (SD = 10.46), a mean BMI of 24.90 (SD = 5.97) and a mean ULS score of 47.86 (SD = 7.93). The majority of participants were unemployed, using SGA, reported no tobacco and alcohol consumption, and exhibited low PA level. Detailed results of the social-demographic and clinical characteristics were presented in Table 1.

**Table 1:** Social-demographic and clinical characteristics of participants

Characteristics	Total (*n* = 538)
Age (years)	44.70 ± 11.45
Gender, n (%)	
Male	253 (47.0)
Female	285 (53.0)
Employment status, n (%)	
Yes	127 (23.6)
No	411 (76.4)
Education levels, n (%)	
Primary education or below	193 (35.9)
Secondary education	174 (32.3)
Higher education	171 (31.8)
Marriage status, n (%)	
Single	228 (42.4)
Marriage	269 (50.0)
Divorced or widowed	41 (7.6)
Tobacco consumption, n (%)	
Yes	85 (15.8)
No	453 (84.2)
Alcohol consumption, n (%)	
Yes	47 (8.7)
No	491 (91.3)
BMI (Kg/m^2^)	24.90 ± 5.97
Illness duration (years)	16.91 ± 10.46
Antipsychotics treatments, n (%)	
None	80 (14.9)
FGA only	23 (4.3)
SGA only	360 (66.9)
Both	75 (13.9)
High waist circumference, n (%)	
Yes	256 (47.6)
No	282 (52.4)
High blood pressure, n (%)	
Yes	227 (42.2)
No	311 (57.8)
Hypertriglyceridemia, n (%)	
Yes	301 (56.0)
No	237 (44.0)
Low-HDL-C, n (%)	
Yes	119 (22.1)
No	419 (77.9)
High fasting glucose, n (%)	
Yes	249 (46.3)
No	289 (53.7)
PA level, n (%)	
Low PA	231 (42.9)
Moderate PA	179 (33.3)
Vigorous PA	128 (23.8)
ULS total score	47.86 ± 7.93
HPLP-C total score	90.51 ± 25.62

Abbreviation: body mass index = BMI, first generation antipsychotics = FGA, second generation antipsychotics = SGA, high-density lipoprotein cholesterol = HDL-C, physical activity = PA, UCLA Loneliness Scale = ULS

### Cluster analysis of HPL

The total HPLP-C score for the 538 participants was 90.51 (SD = 25.62). Participants were divided into two groups using the K-means algorithm. There were 271 participants in the high HPL group with an HPLP-C score of 111.44 (SD = 15.81). There were 267 participants in the low HPL group with an HPLP-C score of 69.27 (SD = 13.11).

### Comparisons between high HPL group and low HPL group

Participants in the high HPL group were more likely to be occupied than those in the low HPL group (29.9% vs. 17.2%, *p* = 0.001). The three education levels between the two groups were significantly different (*p* < 0.001). Participants in the high HPL group had a shorter illness duration than those in the low HPL group (*p* = 0.042). Antipsychotics treatment between the two groups were also significantly different (*p =* 0.003). The proportion of high blood pressure, hypertriglyceridemia and high fasting glucose in the high HPL group were all significantly lower than those in the low HPL group (all *p* < 0.05). In addition, PA levels between the two groups were also significantly different (*p* < 0.001). The high HPL group had a lower level of loneliness when compared with the low HPL group (*p* < 0.001). (see Table 2).

**Table 2:** Differences of social-demographic and clinical characteristics of participants between high HPL group and low HPL group

Characteristics	low HPL group (*n* = 267)	high HPL group (*n* = 271)	χ^2^/t	*P*
Age (years)	45.06 ± 11.31	44.34 ± 11.60	0.733	0.464
Gender, n (%)			0.923	0.337
Male	120 (44.9)	133 (49.1)		
Female	147 (55.1)	138 (50.9)		
Employment status, n (%)			11.955	0.001
Yes	46 (17.2)	81 (29.9)		
No	221 (82.8)	190 (70.1)		
Education levels, n (%)			30.620	< 0.001
Primary education or below	126 (47.2)	67 (24.7)		
Secondary education	76 (28.5)	98 (36.2)		
Higher education	65 (24.3)	106 (39.1)		
Marriage status, n (%)			1.307	0.520
Single	118 (44.2)	110 (40.6)		
Marriage	127 (47.6)	142 (52.4)		
Others	22 (8.2)	19 (7.0)		
Tobacco consumption, n (%)			1.296	0.255
Yes	47 (17.6)	38 (14.0)		
No	220 (82.4)	233 (86.0)		
Alcohol consumption, n (%)			2.645	0.104
Yes	18 (6.7)	29 (10.7)		
No	249 (93.3)	242 (89.3)		
BMI (Kg/m^2^)	24.79 ± 3.90	25.01 ± 7.47	-0.424	0.672
Illness duration (years)	17.84 ± 10.02	16.0 ± 10.82	2.043	0.042
Antipsychotics treatments, n (%)			13.748	0.003
None	54 (20.2)	26 (9.6)		
FGA only	11 (4.1)	12 (4.4)		
SGA only	162 (60.7)	198 (73.1)		
Both	40 (15.0)	35 (12.9)		
High waist circumference, n (%)			1.481	0.224
Yes	120 (44.9)	136 (50.2)		
No	147 (55.1)	135 (49.8)		
High blood pressure, n (%)			31.891	< 0.001
Yes	145 (54.3)	82 (30.3)		
No	122 (45.7)	189 (69.7)		
Hypertriglyceridemia, n (%)			40.454	< 0.001
Yes	186 (69.7)	115 (42.4)		
No	81 (30.3)	156 (57.6)		
Low-HDL-C, n (%)			1.104	0.293
Yes	54 (20.2)	65 (24.0)		
No	213 (79.8)	206 (76.0)		
High fasting glucose, n (%)			6.224	0.013
Yes	138 (51.7)	111 (41.0)		
No	129 (48.3)	160 (59.0)		
PA level, n (%)			62.901	< 0.001
Low PA	158 (59.2)	73 (26.9)		
Moderate PA	74 (27.7)	105 (38.8)		
Vigorous PA	35 (13.1)	93 (34.3)		
ULS total score	50.37 ± 6.43	45.38 ± 8.48	7.694	< 0.001

Abbreviation: health-promoting lifestyle = HPL, body mass index = BMI, first generation antipsychotics = FGA, second generation antipsychotics = SGA, high-density lipoprotein cholesterol = HDL-C, physical activity = PA

### Influencing factors of HPL

The results showed that loneliness (OR = 0.91, 95%CI: 0.89–0.94, *p* < 0.001) was a risk factor for participants to have a high level of HPL. In addition, participants with moderate PA (OR = 2.57, 95%CI: 1.64–4.03, *p* < 0.001) and vigorous PA (OR = 4.77, 95%CI: 2.83–8.02, *p* < 0.001) were protective factors to have a high level of HPL. Participants with secondary education level (OR = 2.15, 95%CI: 1.34–3.43, *p* = 0.001) and higher education level (OR = 2.40, 95%CI: 1.47–3.92, *p* < 0.001) were also protective factors to have a high level of HPL. (See Table 3)

**Table 3:** Logistic regression analyses for variables associated with HPL

Characteristic	OR (95%CI)	*P*
Illness duration	0.99 (0.97, 1.01)	0.268
ULS total score	0.91 (0.89, 0.94)	< 0.001
PA level (ref = low PA)		
Moderate PA	2.57 (1.64, 4.03)	< 0.001
Vigorous PA	4.77 (2.83, 8.02)	< 0.001
Employment status (ref = no employment)	1.50 (0.94, 2.40)	0.091
Antipsychotics treatments (ref = both)		
FGA	1.51 (0.51, 4.47)	0.454
SGA	1.73 (0.95, 3.14)	0.075
None	0.92 (0.43, 1.96)	0.827
Education levels (ref = primary education)		
Secondary education	2.15 (1.34, 3.43)	0.001
Higher education	2.40 (1.47, 3.92)	< 0.001

Abbreviation: health-promoting lifestyle = HPL, UCLA Loneliness Scale = ULS, physical activity = PA, first generation antipsychotics = FGA, second generation antipsychotics = SGA, odds ratios = OR, confidence interval = CI

### Association between HPL and MetS

There were 200 participants in MetS group and 338 participants in non-MetS group. The univariate analysis between MetS group and non-MetS group was shown as table S1. The results of the multivariate logistic regression showed that participants with low HPL level (OR = 2.38, 95%CI: 1.59–3.55, *p* < 0.001) was a risk factor for the prevalence of MetS. (see Table 4)

**Table 4:** Logistic regression analyses of variables associated with MetS

Characteristic	OR (95%CI)	*P*
Age	1.02 (0.99, 1.03)	0.068
gender (ref = male)	1.27 (0.86, 1.87)	0.226
Employment status (ref = no employment)	0.72 (0.45, 1.15)	0.165
BMI	1.17 (1.11, 1.23)	< 0.001
HPL level (ref = high HPL level)	2.38 (1.59, 3.55)	< 0.001
ULS total score	1.01 (0.99, 1.04)	0.415

Abbreviation: metabolic syndrome = MetS, body mass index = BMI, health-promoting lifestyle = HPL, odds ratios = OR, confidence interval = CI

## Discussion

To our knowledge, this is the first study to investigate the influencing factors of HPL among people with schizophrenia and its association with MetS. This study found that loneliness was a risk factor for patients to have a high level of HPL, while engaging in moderate-vigorous physical activity and attaining higher education levels were identified as protective factors. We also found that a low level of HPL was negatively associated with the prevalence of MetS. The findings have important public and clinical implications, in particular for primary healthcare providers.

Our study showed that the loneliness score was high among both of the HPL group. This finding was in line with other studies, which demonstrated that patients with schizophrenia experienced high level of loneliness [1, 11]. Our results also showed loneliness had an adverse impact on patients to achieve a high level of HPL. This finding was similar to a Swiss national health survey [33], which found that lonely participants were prone to unhealthy lifestyle behaviors, such as smoking, being less physically active, and less fruit and vegetable consumption when compared with participants who never felt lonely. A national wide survey among Chinese adults also showed a negative associations between lifestyle scores and loneliness [34]. The reason why lonely individuals are easier to have a poor healthy lifestyle may be related with their poor self-motivation, low spiritual growth, insufficient social support and increased social isolation [35–37]. Previous studies reported that patients with schizophrenia engage less in and make limited use of community resources and had a poorer social relationship [38]. These findings suggest that more attention should be paid on lonely schizophrenia patients, and health-promoting lifestyle interventions in the future should not only focus on reducing negative health behaviors but also consider their impact on alleviating loneliness levels.

The benefits of PA for both physical health and mental health have been well documented [39]. Our finding of the positive association between PA and HPL was in line with the study of Fischer Aggarwal et al. [40], which found that PA could enhance social support, promote a healthy lifestyle, and reduce the risk of CVD. A systematic review and a meta-analysis focused on exploring the associations of sedentary behavior with disease mortality and physical activity level demonstrated a clear dose-response association between sitting time and CVD mortality in inactive population, while moderate intensity physical activity seemed to eliminate the increased risk of death caused by high sitting time [41, 42]. These studies indicated that moderate PA could help people reduce the unhealthy behavior, especially sedentary behavior, which were consistent with our findings. An explanation of this effect is that PA may have positive effects on emotional regulation, stress adaptation, confidence improvement and stay vigorous. However, over half people with schizophrenia in our study failed to meet recommended PA guidelines, such as accruing 150 min of moderate-vigorous PA per week. Our finding was consistent with Seet V et al. [43], who conducted a cross-sectional study in 380 psychiatric patients in Singapore and found a high prevalence of inadequate physical activity (43.2%). This finding indicated a large gap of PA in patients with schizophrenia. Previous studies also showed the effectiveness of PA on health management. An 18-month prospective study involving people with bipolar disorder indicated that physically active patients had lower levels of anxiety and less insomnia [44]. All these findings strongly support the notion that promotion of moderate-vigorous physical activity is an important health promotion task in patients with schizophrenia.

Education is an important social determinant of health that impact health behaviors. Our findings align with previous research that reported a positive association between higher education levels and healthier lifestyle behaviors. A population-based survey conducted in Girona showed that individuals with a lower education level had more lifestyle-related factors of CVD when compared to those with a higher education level [45]. A survey conducted in 52,029 rural Japanese individuals indicated that individuals with higher education level had a lower BMI in women, and more exercise in individuals younger than 70 years [46]. Previous studies have also showed positive effects of education intervention on improving healthy lifestyle. In a study of Du L and Hu J [47], a 5-week health education intervention among 35 Chinese elder without Alzheimer’s disease (AD) led to an increased AD-related knowledge and improved HPL. A randomized controlled study conducted in the perinatology clinic showed that women with gestational diabetes mellitus who received the health-promoting lifestyle education program had a greater improvement of the healthy lifestyle behaviors and quality of life [48]. People with higher levels of education exhibit a greater likelihood of seeking, understanding, and assessing health-related information, making them more prone to actively engage in health-promoting actions [49]. Therefore, it is important for primary healthcare providers to offer education to people with schizophrenia to improve their health-related knowledge, promote healthy lifestyles, and to prevent or ameliorate MetS.

In our study, we observed that the overall prevalence of MetS among schizophrenia patients was 37.17%, consistent with the pooled MetS prevalence reported in a meta-analysis [50]. The findings highlight elevated rates of MetS in individuals with schizophrenia. We also found a negative association between HPL and MetS. This finding was in line with other studies. Garralda-Del-Villar M et al. [51] conducted a 6-year cohort study on people without MetS to explore the relationship between health lifestyle and MetS incidence. Their findings revealed that higher adherence to a healthy lifestyle corresponded to a lower risk of developing MetS. Similarly, Park YS et al. [52] investigated lifestyle factors among 6995 South Korean adults and found that an increased number of lifestyle risk factors was associated with a higher risk of MetS. One study with 225 Latinos illustrated the HPLP sub-score of physical activity contributed to the risk for MetS [53]. Another study with 1128 individuals with MetS also showed physical activity was the strongest predictor of health-promoting behaviors to improve the lifestyle of patients with MetS [54]. Hence, it is clear that physical activity is an important content of healthy lifestyle and should be given a greater emphasis and be implemented in planning and interventions to reduce the risk of MetS.

### Limitations

This study has several limitations that can guide future research in this domain. First, our study employed a non-probability sampling approach due to the specific characteristics of the target population and resource limitations. However, the sampling method may introduce potential selection biases and limit the generalization of our findings although we take multiple strategies to mitigate potential biases, such as established clear inclusion and exclusion criteria to define the target criteria, recruited participants from multiple centers, and provided detailed participants characteristics. External validity of our results across different populations and settings will provide valuable insights into the generalizability of findings. Second, the cross-sectional design makes it impossible to evaluate the causality between health promotion lifestyle and factors including loneliness, education literacy and physical activity. Third, our study used ICD-10 for diagnosing schizophrenia, which may reflect the temporal constraints existing at the initiation of data collection, predating the availability of ICD-11 and DSM-5™. Future research should consider adopting the latest diagnostic criteria for improved alignment with current standards. Forth, this study has no healthy control group, it is difficult to understand the health status gap between patients with schizophrenia and general population.

## Conclusions

Our findings suggest that HPL may be a potentially straightforward tool for reducing MetS for patients with schizophrenia. Understanding the influencing factors associated with HPL will provide direction for determining effective strategies to promote healthy lifestyle behaviors and enhance physical health for this population. The results of our findings indicate that health education related to HPL could be offered in primary health centers to help patients improve their health literacy. Primary healthcare professionals are encouraged to devote more attention to promote HPL in patients with schizophrenia and to motivate them to adopt more positive lifestyle choices, such as engaging in moderate-vigorous physical activities.

### Electronic supplementary material

Below is the link to the electronic supplementary material.

Supplementary Material 1

## Data Availability

The datasets generated and/or analyzed during the current study are not publicly available due to confidentiality in the informed consent. However, the datasets are available from the corresponding author upon reasonable request.

## Abbreviations

MetS: Metabolic syndrome
BMI: Body mass index
FGA: First generation antipsychotics
SGA: Second generation antipsychotics
HDL-C: High-density lipoprotein cholesterol
HPL: Health-promoting lifestyle
PA: Physical activity
ULS: UCLA Loneliness Scale
