# Supplementary material for: Determinants and outcomes of health-promoting lifestyle among people with schizophrenia
Source: BMC Psychiatry. 2024 Mar 4;24:177. doi: 10.1186/s12888-024-05625-2 (PMC10913642; doi:10.1186/s12888-024-05625-2)
Supplement: Supplementary file 1 — Supplementary Material 1 [file 12888_2024_5625_MOESM1_ESM.docx]

Supplementary material of Determinants and outcomes of health-promoting lifestyle among people with schizophrenia

Table S1 Differences of social-demographic and clinical characteristics of participants between MetS group and non-MetS group

| Characteristics | non-MetS group  (n= 338 ) | MetS group  (n = 200) | *χ^2^/t* | *P* |
| --- | --- | --- | --- | --- |
| Age (years) | 43.89 ± 11.52 | 46.06 ± 11.23 | -2.126 | 0.034 |
| Gender, n (%) |  |  | 5.442 | 0.020 |
| Male | 172 (50.9) | 81 (40.5) |  |  |
| Female | 166 (49.1) | 119 (59.5) |  |  |
| Employment status, n (%) |  |  | 7.703 | 0.006 |
| Yes | 93 (27.5) | 34 (17.0) |  |  |
| No | 245 (72.5) | 166 (83.0) |  |  |
| Education levels, n (%) |  |  | 3.648 | 0.161 |
| Primary education or below | 111 (32.8) | 82 (41.0) |  |  |
| Secondary education | 114 (33.8) | 60 (30.0) |  |  |
| Higher education | 113 (33.4) | 58 (29.0) |  |  |
| Marriage status, n (%) |  |  | 2.143 | 0.343 |
| Single | 147 (43.5) | 81 (40.5) |  |  |
| Marriage | 162 (47.9) | 107 (53.5) |  |  |
| Divorced or widowed | 29 (8.6) | 12 (6.0) |  |  |
| Tobacco consumption, n (%) |  |  | 0.404 | 0.525 |
| Yes | 56 (16.6) | 29 (14.5) |  |  |
| No | 282 (83.4) | 171 (85.5) |  |  |
| Alcohol consumption, n (%) |  |  | 1.203 | 0.273 |
| Yes | 33 (9.8) | 14 (7.0) |  |  |
| No | 305 (90.2) | 186 (93.0) |  |  |
| BMI (Kg/m^2^) | 23.86 ± 3.75 | 26.66 ± 8.20 | -4.550 | ＜0.001 |
| Illness duration (years) | 16.44 ± 10.28 | 17.71 ± 10.74 | -1.366 | 0.173 |
| Antipsychotics treatments, n (%) |  |  | 4.864 | 0.182 |
| None | 43 (12.7) | 37 (18.5) |  |  |
| FGA | 13 (3.9) | 10 (5.0) |  |  |
| SGA | 237 (70.1) | 123 (61.5) |  |  |
| Both | 45 (13.3) | 30 (15.0) |  |  |
| High waist circumference, n (%) |  |  | 67.029 | ＜0.001 |
| Yes | 115 (34.0) | 141 (70.5) |  |  |
| No | 223 (66.0) | 59 (29.5) |  |  |
| High blood pressure, n (%) |  |  | 112.100 | ＜0.001 |
| Yes | 84 (24.8) | 143 (71.5) |  |  |
| No | 254 (75.2) | 57 (28.5) |  |  |
| Hypertriglyceridemia, n (%) |  |  | 116.652 | ＜0.001 |
| Yes | 129 (38.2) | 172 (86.0) |  |  |
| No | 209 (61.8) | 28 (14.0) |  |  |
| Low-HDL-C, n (%) |  |  | 76.763 | ＜0.001 |
| Yes | 34 (10.1) | 85 (42.5) |  |  |
| No | 304 (89.9) | 115 (57.5) |  |  |
| High fasting glucose, n (%) |  |  | 141.285 | ＜0.001 |
| Yes | 90 (26.6) | 159 (79.5) |  |  |
| No | 248 (73.4) | 41 (20.5) |  |  |
| HPL level, n (%) |  |  | 24.504 | ＜0.001 |
| Low HPL | 140 (41.4) | 127 (63.5) |  |  |
| High HPL | 198 (58.6) | 73 (36.5) |  |  |
| PA level, n (%) |  |  | 1.442 | 0.486 |
| Low PA | 139 (41.1) | 92 (46.0) |  |  |
| Moderate PA | 118 (34.9) | 61 (30.5) |  |  |
| Vigorous PA | 81 (24.0) | 47 (23.5) |  |  |
| ULS total score | 47.25 ± 8.26 | 48.88 ± 7.23 | -2.388 | 0.017 |

Abbreviation: metabolic syndrome=MetS, body mass index=BMI, first generation antipsychotics=FGA, second generation antipsychotics=SGA, high-density lipoprotein cholesterol=HDL-C, health-promoting lifestyle=HPL, physical activity=PA
